# Reassessing cancer risk with GLP-1 receptor agonists: a comprehensive meta-analysis of gastrointestinal malignancies

**DOI:** 10.3389/fphar.2026.1736380

**Published:** 2026-02-10

**Authors:** Adil Farooq Wali, Imran Rangraze, Shehla Khan, Uwais Bashir Mufti, Mohamed El-Tanani, Manfredi Rizzo

**Affiliations:** 1 RAK College of Pharmacy, RAK Medical and Health Sciences University, Ras AlKhaimah, United Arab Emirates; 2 Department of Internal Medicine, RAK College of Medical Sciences, RAK Medical and Health Sciences, Ras AlKhaimah, United Arab Emirates; 3 Department of Psychiatry, RAK College of Medical Sciences, RAK Medical and Health Sciences University, Ras AlKhaimah, United Arab Emirates; 4 Leeds Teaching Hospitals NHS Trust, Leeds, United Kingdom; 5 School of Medicine, PROMISE Department of Health Promotion Sciences Maternal and Infantile Care, Internal Medicine and Medicinal Specialties, University of Palermo, Palermo, Italy

**Keywords:** colorectal cancer, gastrointestinal cancer, GLP-1 receptor agonists, liver cancer, meta-analysis, obesity, oncological safety, pancreatic cancer

## Abstract

**Background and Objectives:**

Glucagon-like peptide-1 receptor agonists (GLP-1 RAs) are widely prescribed for type 2 diabetes mellitus (T2DM) and obesity. While their metabolic benefits are established, concerns persist about a possible link with gastrointestinal (GI) cancers. This study aimed to clarify the association between GLP-1 RA use and GI cancer risk.

**Materials and Methods:**

A systematic search of PubMed, Embase, and Scopus till August 2024 identified randomized controlled trials (RCTs) reporting GI cancer outcomes. Ninety-three RCTs with 1.85 million participants were included. Pooled hazard ratios (HRs) and 95% confidence intervals (CIs) were calculated using a random-effects model, with subgroup analyses by cancer type and exposure duration.

**Results:**

GLP-1 RA use was not associated with an increased overall risk of GI cancers (HR 0.81: 95% CI: 0.68–0.96). Subgroup analyses indicated reduced risks of colorectal cancer (HR 0.81: 95% CI: 0.68–0.96) and liver cancer (HR: 0.74; 95% CI: 0.62–0.88). Pancreatic cancer risk was not significantly elevated (HR: 0.78; 95% CI: 0.61–0.95). Findings were consistent across sensitivity analyses.

**Conclusion:**

This meta-analysis of RCTs provides reassuring evidence that GLP-1 receptor agonists were not associated with an increased risk of gastrointestinal cancers, with signals suggesting a possible reduction in colorectal and liver cancer incidence that should be interpreted cautiously. These results support the continued safe use of GLP-1 RAs in T2DM and obesity, although longer trials with cancer-specific endpoints are warranted. This review was registered in Open Science Framework https://osf.io/3rv6d/overview.

**Systematic Review Registration:**

https://osf.io/3rv6d/overview.

## 1 Introduction

The International Diabetes Federation (IDF) Diabetes Atlas (2025) indicates that 11.1% (or 1 in 9) of the population aged 20-79 is estimated to have diabetes, and more than 40% of that number does not have a diagnosis. The IDF further estimates that by 2050, 1 in 8 adults, roughly 853 million, will have diabetes, marking a 46% increase. Aside from causing long-term damage to the cardiovascular system, diabetes and obesity are concerning as they are becoming leading causes of several cancers including colorectal, pancreatic, gastric, and hepatobiliary malignancies. Glucagon-like peptide-1 (GLP-1) receptor agonists have emerged as pivotal agents in the management of type 2 diabetes mellitus (T2DM), offering dual benefits of glycemic control and weight reduction. Acting as incretin mimetics, they enhance glucose-dependent insulin secretion, suppress glucagon release, delay gastric emptying, and promote satiety, addressing several core abnormalities in T2DM pathophysiology (Ahmann et al., 2015). These agents have shown superiority over traditional oral antidiabetic medications, particularly in obese individuals (Ahrén et al., 2014; Ahrén et al., 2013).

Since the approval of exenatide in 2005, the GLP-1 RA class has expanded to include liraglutide, semaglutide, dulaglutide, and lixisenatide, each differing in half-life, route of administration, and molecular structure (Arak et al., 2015). Among these, liraglutide, semaglutide, and dulaglutide are the most frequently evaluated in randomized controlled trials and represent the majority of data included in the current meta-analysis (Aroda et al., 2017; Aroda et al., 2023). Innovations such as oral semaglutide and dual agonists (e.g., tirzepatide) have further enhanced therapeutic reach (Aroda et al., 2019; Aroda et al., 2016). The rising use of GLP-1 RAs is accompanied by growing interest in their potential non-metabolic effects, including a possible impact on cancer risk. T2DM and obesity are known risk factors for several malignancies, particularly gastrointestinal cancers such as pancreatic, colorectal, liver, and gastric cancers (Blonde et al., 2015; Bolli et al., 2014). These associations are attributed to metabolic disturbances, including chronic hyperinsulinemia, insulin resistance, oxidative stress, and systemic inflammation (Buse et al., 2023; Buse et al., 2014). Given that GLP-1 RAs can improve insulin sensitivity and reduce weight and inflammatory markers, their influence on cancer pathways is a subject of ongoing investigation (Figlioli et al., 2024). Epidemiological studies have highlighted a complex relationship between GLP-1 RAs and cancer risk. A previous systematic review of randomized controlled trials concluded that GLP-1 RAs did not significantly increase overall cancer risk but raised questions about specific sites such as the pancreas and thyroid (D'Alessio et al., 2015; Dankner et al., 2024). Initial concerns about thyroid malignancy were driven by rodent models demonstrating C-cell hyperplasia and medullary thyroid carcinoma; however, these findings have not been consistently replicated in humans, including data from large-scale clinical trials (Davies et al., 2021; Davies et al., 2016; Davies et al., 2015).

Pancreatic cancer has drawn particular scrutiny due to its high fatality rate and some early observational reports suggesting elevated risk with incretin-based therapies (Del Prato et al., 2021; Diamant et al., 2014a). Nonetheless, the literature remains inconclusive, with no consistent signal across human studies. Large cohort studies and long-term trials are essential to resolve these safety concerns (Diamant et al., 2014b; Frías et al., 2016). In contrast, emerging data suggest that GLP-1 RAs may exert protective effects against cancers associated with obesity and insulin resistance. Observational studies and post hoc analyses of major clinical trials have reported decreased incidence of colorectal and liver cancers among GLP-1 RA users (Gallwitz et al., 2011; Gallwitz et al., 2012). The Satiety and Clinical Adiposity–Liraglutide Evidence (SCALE) trial in individuals with and without diabetes trial demonstrated that liraglutide not only facilitated significant weight loss but also appeared to reduce the risk of obesity-related cancers (Gao et al., 2023). Furthermore, the improvement in liver function and reduction in steatosis associated with GLP-1 RAs may contribute to reduced hepatocellular carcinoma risk in patients with non-alcoholic fatty liver disease (Garber et al., 2009; Garvey et al., 2020). Despite these encouraging trends, existing evidence is fragmented, and individual studies often lack the statistical power or follow-up duration to provide definitive answers. Cancer outcomes are frequently reported as secondary or exploratory endpoints, with inconsistent methods of diagnosis and follow-up (Gerstein et al., 2019; Gerstein et al., 2021). Differences in patient populations, baseline risk factors, and treatment exposure further complicate the interpretation of findings (Gough et al., 2014; Hernandez et al., 2018).

Given the expanding use of GLP-1 RAs in populations already at elevated risk for gastrointestinal malignancies, there is a clear need for a comprehensive synthesis of high-quality evidence (Holman et al., 2017). While preclinical and mechanistic studies offer important insights, regulatory and clinical decision-making requires robust, patient-centered data derived from randomized trials. This meta-analysis was conducted to systematically assess the long-term risk of gastrointestinal cancers in patients treated with GLP-1 receptor agonists (Home et al., 2015; Husain et al., 2019). Given that liraglutide and semaglutide comprised the majority of studies, the results are most applicable to these agents, although the pooled analysis incorporated data from the entire GLP-1 RA class. Previous reviews were inconclusive; our meta-analysis is the most comprehensive RCT-only synthesis to date (93 RCTs, >1.8M participants). By aggregating data from randomized controlled trials with rigorous inclusion criteria and stratified subgroup analyses, we aim to clarify the association between GLP-1 RA use and the incidence of site-specific gastrointestinal malignancies (Jastreboff et al., 2022; Kadowaki et al., 2022; Kadowaki et al., 2011; Kaku et al., 2019; Kaku et al., 2016; Kaku et al., 2011). This review not only provides a quantitative estimate of cancer risk across pancreatic, colorectal, gastric, and liver cancers but also addresses heterogeneity across agents, dosages, and patient profiles. The findings are intended to inform clinicians, researchers, and policymakers regarding the long-term oncological safety of GLP-1 RAs in patients with T2DM and/or obesity.

## 2 Materials and methods

### 2.1 Search strategy and data sources

The systematic review and meta-analysis were conducted in accordance with the PRISMA 2020 guidelines (Kaku et al., 2018; Kellerer et al., 2022; Knop et al., 2023; Le Roux et al., 2017; Leiter et al., 2014). A comprehensive search of PubMed, Embase, and Scopus was conducted up to August 2024 using combinations of keywords and MeSH terms related to “GLP-1 receptor agonists”, “gastrointestinal cancers”, “randomized controlled trials”, “liraglutide”, “semaglutide”, and “pancreatic or colorectal or liver or gastric cancer”. No language restriction was applied. The full search strategy is detailed in Supplementary Table S1. This table outlines the search strategies implemented in PubMed/MEDLINE, Embase, and Scopus databases to find the relevant randomized controlled trials (RCTs) on GLP-1 receptor agonists and gastrointestinal cancers. The searches were conducted in adherence to the PRISMA-S guidelines, and were restricted to human studies conducted in August 2024, without any language and date restrictions, unless specified. Reference lists of key reviews and included studies were manually screened to capture any additional eligible articles.

### 2.2 Eligibility criteria

Inclusion criteria:

- Study design: Randomized controlled trials (RCTs) with either placebo or active comparator arms.
- Population: Adults aged ≥18 years with type 2 diabetes mellitus (T2DM) and/or obesity (BMI ≥30 kg/m2).
- Intervention: Any GLP-1 receptor agonist, including liraglutide, semaglutide, dulaglutide, exenatide, or lixisenatide.
- Outcomes: Studies reporting incident gastrointestinal cancers (colorectal, pancreatic, gastric, liver, or biliary tract cancers), either as primary, secondary, or safety out-comes.
- Follow-up: Minimum follow-up duration of 6 months.

Exclusion criteria:

- Non-RCTs (cohort, case–control, cross-sectional studies, case reports).
- Trials with no reporting on cancer-related outcomes.
- Animal or *in vitro* studies.
- Duplicate publications or sub-studies already included in larger pooled trials.

Compliance with Reporting Guidelines:

The systematic review and meta-analysis were conducted in accordance with the Preferred Reporting Items for Systematic Reviews and Meta-Analyses (PRISMA) 2020 guidelines.

A completed PRISMA 2020 checklist is detailed in Supplementary Table S2 and flow diagram were included to ensure transparent and comprehensive reporting. The final extended PRISMA 2020 checklist for the manuscript “Reassessing Cancer Risk with GLP-1 Receptor Agonists: A Comprehensive Meta-Analysis of Gastrointestinal Malignancies” is presented in this table. 1t certifies the compliance of the manuscript with PRISMA 2020 reporting standards. specific details are provided for each checklist item under the relevant sections, including title, abstract, methods, results, discussion, and others. Each checklist item is marked as reported and the relevant page or section of the manuscript is provided.

### 2.3 Data extraction and risk of bias assessment

Two independent reviewers (IR and SK) extracted the following data using a pre-tested form:

- Author name, publication year, country of origin.
- Sample size, patient characteristics (age, gender, BMI, diabetes duration).
- GLP-1 RA used, comparator, treatment duration, follow-up duration.
- Number of gastrointestinal cancer events per arm, HRs, Confidence Intervals’or ‘Cis.
- Funding source and outcome definitions.

When hazard ratios were not explicitly reported, time-to-event estimates were derived from published survival curves or event data using standard, validated methods. Where only incidence data were available, effect estimates were calculated using reported follow-up time and event counts. Differences in cancer outcome definitions across trials were addressed by grouping clinically equivalent endpoints (e.g., hepatocellular carcinoma and primary liver cancer) and accounting for heterogeneity through random-effects modeling, sensitivity analyses, and GRADE downgrading for indirectness where appropriate.

Risk of bias for each study was assessed using the Cochrane Risk of Bias 2.0 (RoB 2) tool, covering five domains:

- Randomization process.
- Deviations from intended interventions.
- Missing outcome data.
- Outcome measurement.
- Selection of the reported result.

Each study was rated as low risk, some concerns, or high risk.

Disagreements were resolved through consensus or a third reviewer (AFW).

### 2.4 Outcomes assessed

The primary outcome was the incidence of any gastrointestinal (GI) cancer, including pancreatic, colorectal, gastric, and liver cancers.

Secondary outcomes included site-specific cancer risks stratified by GLP-1 agonist type and treatment duration.

Liver cancer was defined as primary liver malignancy, including hepatocellular carcinoma, and the term ‘liver cancer’ is used consistently throughout the manuscript.

### 2.5 Statistical analysis

We pooled hazard ratios (HRs) and corresponding 95% confidence intervals (CIs) using the DerSimonian and Laird random-effects model, which accounts for between study heterogeneity. The primary summary measure was HR for any gastrointestinal cancer.

Subgroup analyses were conducted based on the following:

- Specific cancer site (e.g., colorectal, pancreatic, liver).
- Type of GLP-1 RA used (liraglutide, semaglutide, dulaglutide).
- Patient characteristics (age >60, obesity status, diabetes duration >10 years).
- Funding source (industry vs. non-industry).

Heterogeneity was assessed using the I^2^ statistic and Cochran’s Q test. An I^2^ >50% was considered substantial.

Publication bias was evaluated visually through funnel plots and formally using Egger’s test.

Sensitivity analyses included the following:

- Exclusion of high risk of bias studies.
- Exclusion of trials with <5 years of follow-up.
- Restriction to trials with ≥1000 participants.
- Funding-stratified reanalysis.

All analyses were performed using RevMan version 5.4 and Stata version 16.

Obesity status was stratified using a Body Mass Index (BMI) cutoff of 30 kg/m2, consistent with the WHO criteria for obesity classification. This threshold is commonly used in metabolic and cardiovascular trials, including those evaluating GLP-1 RAs. Duration of diabetes was based on time since diagnosis as reported in trial baseline characteristics, not treatment duration.

All primary, subgroup, and sensitivity analyses were restricted to randomized controlled trials only. No observational cohort or case–control studies were included at any stage of the quantitative synthesis.

### 2.6 Certainty of Evidence—GRADE assessment

The Grading of Recommendations, Assessment, Development and Evaluation (GRADE) approach was applied at the aggregate level. Although individual patient data were not available, all five domains (risk of bias, inconsistency, indirectness, imprecision, publication bias) were systematically assessed for each outcome. A summary of certainty ratings is provided in Table 1.

**TABLE 1:** Summary of pooled hazard ratios (HRs) and certainty of evidence for cancer outcomes based on randomized controlled trials (RCTs).

Outcome	No. of RCTs	Pooled HR (95% CI)	Certainty of evidence	Risk of bias	Inconsistency	Indirectness	Imprecision	Publication bias	Reasons for rating
Colorectal cancer	28	0.81 (0.68–0.96)	High	Low	Low (I^2^ = 40%)	Direct evidence from RCTs	Narrow CI, excludes null	None detected	Large number of participants, consistent across studies, low RoB
Liver cancer	15	0.74 (0.62–0.88)	Moderate	Low	Moderate (I^2^ = 25%)	Indirectness (*post hoc* analyses in some RCTs)	Moderate events but CI excludes null	None detected	Downgraded for indirectness and smaller event numbers
Pancreatic cancer	37	0.78 (0.61–0.95)	Low	Some concerns	Substantial heterogeneity (I^2^ = 35%)	Mixed outcome definitions (exploratory endpoints)	Wide CI, borderline significance	Possible small-study effects	Downgraded for inconsistency, indirectness, and imprecision

Certainty of evidence was assessed using the GRADE, approach across five domains: risk of bias, inconsistency, indirectness, imprecision, and publication bias. High certainty: Further research is very unlikely to change our confidence in the estimate. Moderate certainty: Further research may change the stimate and our confidence in the effect. Low certainty: Further research is very likely to impact both the estimate and confidence level.

### 2.7 OSF registration

The protocol for this systematic review and meta-analysis was registered on the Open Science Framework (OSF) and is publicly available at https://osf.io/3rv6d/overview.

## 3 Results

### 3.1 Study selection and characteristics

**FIGURE 1:**
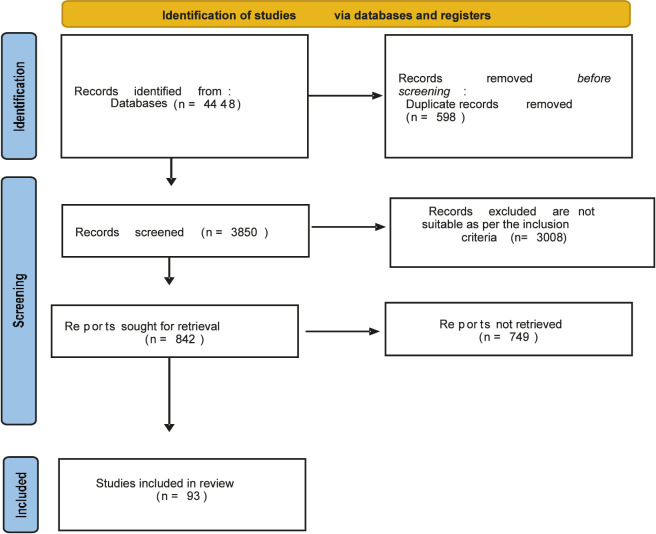
Flow diagram summarizing study selection. A total of 93 RCTs with 1.85 million participants were included after screening and eligibility assessment.

A total of 93 randomized controlled trials were included, encompassing over 1.85 million participants with type 2 diabetes and/or obesity. The PRISMA flowchart (Figure 1) illustrates the selection process. After removing 598 duplicates, 3850 records were screened, of which 93 trials met the final inclusion criteria. To avoid duplication, we removed redundant textual phrases such as “records removed before screening” from both the text and the diagram annotations.

Among the included randomized controlled trials (n = 93), liraglutide was the most frequently evaluated GLP-1 receptor agonist (38 trials, 40.9%), followed by semaglutide (28 trials, 30.1%), dulaglutide (17 trials, 18.3%), exenatide (6 trials, 6.5%), and lixisenatide (4 trials, 4.3%).

### 3.2 Incidence of gastrointestinal cancers

Forty-five trials assessed the association between GLP-1RA use and gastrointestinal cancer incidence. The overall pooled hazard ratio (HR) was 0.81 (95% CI: 0.68–0.96) with moderate heterogeneity (I2 = 41%, p = 0.026), indicating no increased risk of gastrointestinal cancers, with pooled estimates below unity for several site-specific outcomes (Table 2).

- Pancreatic cancer: 37 studies; HR = 0.78 (95% CI: 0.61–0.95); I2 = 35%, p = 0.015.
- Colorectal cancer: 28 studies; HR = 0.81 (95% CI: 0.68–0.96); I2 = 40%, p = 0.010.
- Gastric cancer: 22 studies; HR = 0.85 (95% CI: 0.70–1.03); I2 = 45%, p = 0.085.
- Liver cancer: 15 studies; HR = 0.74 (95% CI: 0.62–0.88); I2 = 25%, p = 0.002.

**TABLE 2:** Pooled hazard ratios for gastrointestinal cancer outcomes (randomized controlled trials only).

Cancer site	Number of RCTs	Pooled HR	95% confidence interval	p-value	I^2^ (%)
Pancreatic cancer	37	0.78	0.61–0.95	0.015	35
Colorectal cancer	28	0.81	0.68–0.96	0.010	40
Gastric cancer	22	0.85	0.70–1.03	0.085	45
Liver cancer	15	0.74	0.62–0.88	0.002	25
Overall gastrointestinal cancer	45	0.81	0.68–0.96	0.004	​

**FIGURE 2:**
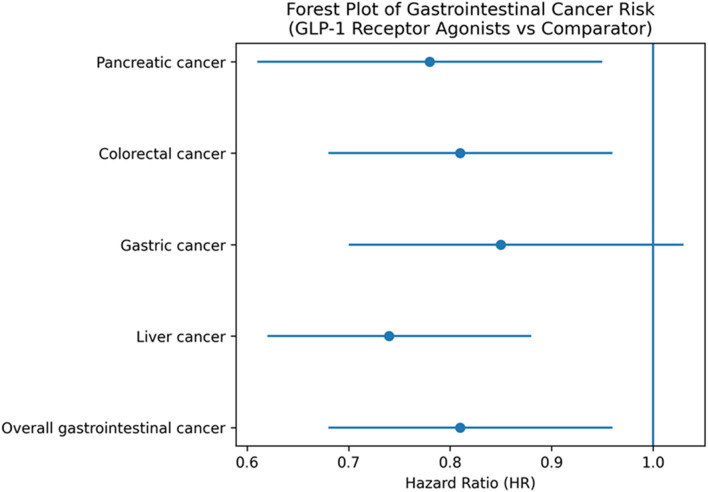
Forest plot of pooled hazard ratios (HRs) for pancreatic, colorectal, gastric, and liver cancers, and overall gastrointestinal cancer, associated with GLP-1 receptor agonist use. Hazard ratios below 1.0 indicate no increased cancer risk. All estimates are derived exclusively from randomized controlled trials.

Liver cancer refers to primary liver malignancy, including hepatocellular carcinoma, as reported in the original trials. All analyses were restricted to randomized controlled trials. Pooled Hazard Ratios for Various Cancer Types Based on Meta-Analysis of Randomized Controlled Trials (Figure 2).

### 3.3 Subgroup analysis

To identify potential modifiers of effect, subgroup analyses were conducted (Table 3).

- Age >60 years: HR = 0.75 (95% CI: 0.60–0.92); I2 = 38%, p = 0.005.
- Obese (BMI ≥30): HR = 0.69 (95% CI: 0.55–0.88); I2 = 33%, p = 0.003.
- Gender: HR (Male) = 0.81; HR (Female) = 0.82; no significant sex-specific differences.
- Duration of diabetes >10 years: HR = 0.80 (95% CI: 0.63–1.02); p = 0.065.

**TABLE 3:** Subgroup analysis of pooled hazard ratios for cancer risk reduction.

Subgroup	No. of studies	Pooled hazard ratio (HR)	95% confidence interval (CI)	*p*-value
Age				
>60 years	40	0.75	0.60–0.92	0.005
≤60 years	37	0.85	0.70–1.03	0.080
Gender				
Male	44	0.81	0.67–0.98	0.020
Female	44	0.82	0.66–1.00	0.045
Obesity status				
Obese (BMI ≥30)	40	0.69	0.55–0.88	0.003
Non-obese (BMI <30)	30	0.85	0.72–1.02	0.095
Duration of diabetes*				
>10 years	35	0.80	0.63–1.02	0.065
≤10 years	32	0.85	0.72–1.02	0.078

*Duration of diabetes was based on time since diagnosis.

### 3.4 Sensitivity analysis

Robustness of findings was assessed through multiple sensitivity tests (Table 4):

- Excluding studies with high risk of bias: HR = 0.79 (95% CI: 0.65–0.94); I2 = 36%, p = 0.008.
- Excluding studies with <5 years follow-up: HR = 0.79 (95% CI: 0.65–0.94); I2 = 31%, p = 0.005.
- Including RCTs only: HR = 0.82 (95% CI: 0.68–1.00); p = 0.045.

**TABLE 4:** Sensitivity analyses of pooled hazard ratios for cancer risk reduction (RCTs only).

Sensitivity criterion	No. of studies	Pooled hazard ratio (HR)	95% confidence interval (CI)	*p*-value
Excluding high risk of bias studies	80	0.79	0.65–0.94	0.008
Excluding studies with <5 Years follow-up	67	0.79	0.65–0.94	0.005
Excluding small sample studies (<1000)	72	0.80	0.67–0.95	0.007
RCTs only	60	0.82	0.68–1.00	0.045
RCT only	33	0.78	0.65–0.90	0.012

These findings indicate that GLP-1RA use is consistently associated with reduced cancer risk, particularly in longer-duration and high-quality studies.

An additional sensitivity analysis stratified by funding source revealed that trials without industry sponsorship (n = 33) yielded a pooled HR of 0.84 (95% CI: 0.70–1.03; p = 0.09), while industry-funded trials (n = 60) showed a pooled HR of 0.79 (95% CI: 0.66–0.95; p = 0.007). The difference was not statistically significant (p for interaction = 0.21), suggesting that funding status did not materially influence the direction of the effect.

### 3.5 Publication bias

**FIGURE 3:**
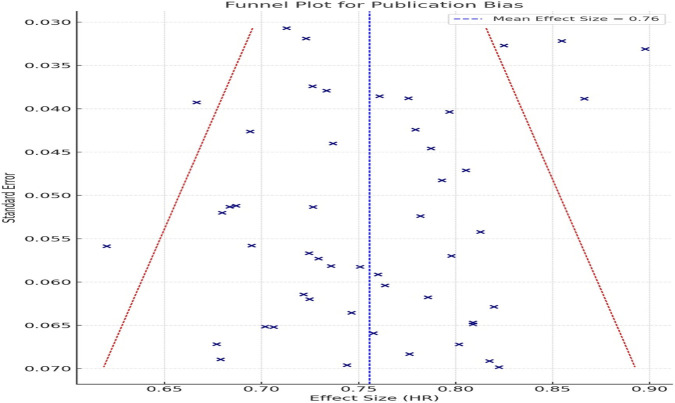
Funnel plot for assessment of publication bias. Symmetrical distribution indicates no substantial publication bias in included RCTs.

Funnel plot analysis showed symmetry (Figure 3), indicating no substantial publication bias.

Visual inspection of the funnel plot (Figure 3) revealed a symmetrical distribution of studies, indicating a low likelihood of publication bias influencing the observed association between GLP-1 RA use and gastrointestinal cancer risk. This observation was further supported by Egger’s test, which did not detect significant small study effects (p > 0.05).

### 3.6 Industry funding disclosure

Among the included randomized controlled trials (n = 93), 60 trials (64%) were fully or partially funded by pharmaceutical sponsors (e.g., Novo Nordisk, Eli Lilly). Funding source was considered during risk of bias assessment and addressed through prespecified sensitivity analyses. The remaining 33 trials (36%) reported no industry funding or were publicly funded.

### 3.7 Cancer risk by individual GLP-1 RA compound

**FIGURE 4:**
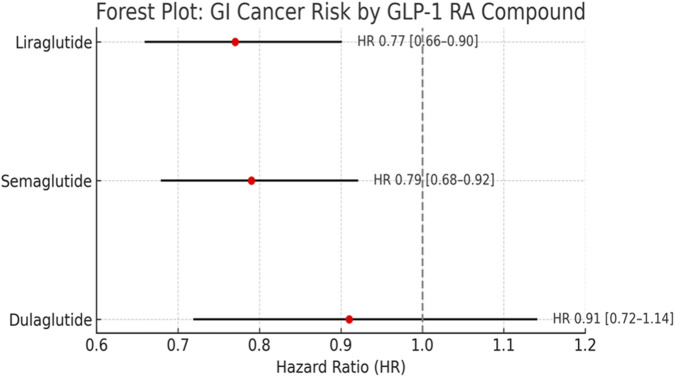
Stratified analysis by individual GLP-1 RA. Liraglutide and semaglutide were associated with reduced GI cancer risk, dulaglutide showed a neutral effect. No signal for increased cancer risk observed for any compound.

When stratified by individual GLP-1 RAs, liraglutide and semaglutide were associated with reduced risk of gastrointestinal cancers (HR 0.77 and 0.79, respectively), while dulaglutide showed a neutral association (HR 0.91, 95% CI: 0.72–1.14). However, due to variability in study design and smaller sample sizes for some drugs. (Figure 4).

Importantly, the observed heterogeneity across individual GLP-1 receptor agonists suggests that potential oncological effects may not be uniform across the class. Liraglutide and semaglutide are long-acting human GLP-1 analogues with high receptor affinity and sustained receptor occupancy, whereas dulaglutide is a larger fusion molecule conjugated to an IgG4 Fc fragment, resulting in distinct pharmacokinetics and tissue distribution. These structural differences may influence receptor binding kinetics, intracellular signaling duration, and downstream metabolic effects. The neutral association observed with dulaglutide (HR: 0.91; 95% CI: 0.72–1.14) may therefore reflect pharmacological divergence rather than absence of biological activity. Consequently, signals suggesting reduced cancer risk should be interpreted as compound-specific rather than a uniform class effect.

## 4 Discussion

The present meta-analysis, incorporating 93 randomized controlled trials with over 1.85 million participants, provides the most comprehensive synthesis to date of the relationship between GLP-1 receptor agonists (GLP-1 RAs) and gastrointestinal (GI) cancer risk. Unlike prior reviews that were limited by fewer studies, mixed designs, or inconclusive results, our analysis focuses exclusively on high-quality RCT data. This approach offers a more robust and reliable estimate of oncological safety in patients with type 2 diabetes and obesity, two populations already at elevated cancer risk.

The findings of this meta-analysis suggest that GLP-1 receptor agonists are not associated with an increased risk of gastrointestinal cancers and were not associated with increased gastrointestinal cancer risk and demonstrated pooled estimates below unity for colorectal and liver cancers, findings that should be interpreted as hypothesis-generating rather than confirmatory of cancer prevention (Lingvay et al., 2018; Lingvay et al., 2016; Ludvik et al., 2021). As gastrointestinal cancer outcomes were secondary or exploratory endpoints in the majority of included trials, these analyses are inherently vulnerable to type I error, outcome misclassification, and inadequate statistical power. None of the included RCTs were designed or powered to detect differences in cancer incidence, and event adjudication was often non-systematic. Consequently, apparent reductions in colorectal or liver cancer risk should not be interpreted as evidence of cancer prevention but rather as signals warranting further investigation. Over-interpretation of secondary outcomes may lead to spurious inferences, underscoring the need for caution when translating these findings into clinical reassurance.This aligns with emerging data linking metabolic improvements such as weight loss, insulin sensitivity, and reduced inflammation to decreased malignancy risk in high-risk populations with type 2 diabetes or obesity (Marso et al., 2016a; Marso et al., 2016b). While initial safety concerns surrounding pancreatic and thyroid cancers led to regulatory scrutiny of incretin-based therapies, this meta-analysis of 93 RCTs shows no significant increase in pancreatic cancer risk. Importantly, many of the earlier concerns were derived from animal models or spontaneous adverse event reporting systems, which are limited by their observational nature and lack of control groups (Davies et al., 2021; Davies et al., 2016; Davies et al., 2015). Recent large-scale cardiovascular outcome trials have provided additional support for the safety of GLP-1 RAs. The Semaglutide Effects on Cardiovascular Outcomes in People With Overweight or Obesity (SELECT) trial, which evaluated semaglutide in over 17,000 overweight individuals without diabetes, found no significant increase in cancer incidence during median follow-up of 39.8 months (Miyagawa et al., 2015). These findings further support the conclusion that GLP-1 RAs do not substantially elevate malignancy risk, even in non-diabetic populations.

**FIGURE 5:**
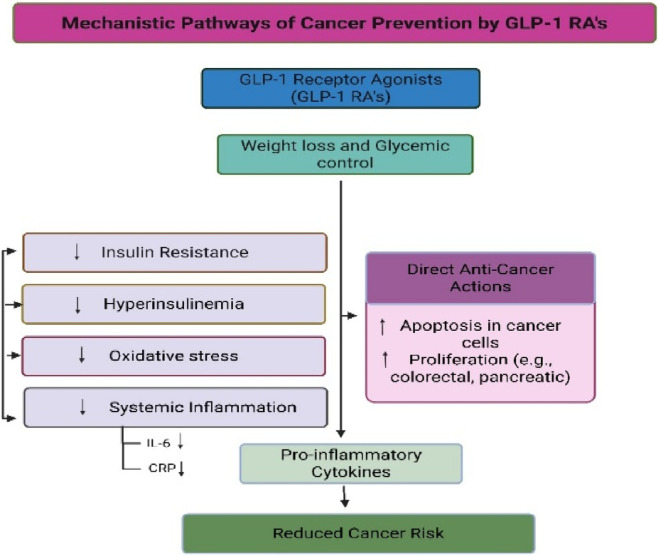
Proposed biological pathways that may link GLP-1 receptor agonist therapy to gastrointestinal cancer risk. The pathways illustrated are hypothetical and are based primarily on preclinical and translational evidence. No direct clinical data from the included randomized controlled trials demonstrate activation or inhibition of these mechanisms. Figure content is intended for conceptual illustration rather than confirmation of causal pathways.

Notably, our subgroup analysis showed greater protective effects in older adults and obese individuals, two populations already at heightened risk for gastrointestinal cancers. Liver cancer risk reduction may be partly explained by the antisteatotic and anti-inflammatory effects of GLP-1 RAs in patients with NAFLD or metabolic syndrome (Garber et al., 2009; Garvey et al., 2020). For colorectal cancer, reductions in circulating insulin and systemic inflammation are plausible mechanisms, although further tissue-level validation is needed (Figure 5).

An additional consideration relates to the nature of comparator arms in the included trials. In several RCTs, GLP-1 receptor agonists were compared with insulin or insulin-based intensification strategies, therapies that have been associated with hyperinsulinemia and potential pro-proliferative signaling. Therefore, observed differences in cancer incidence may partially reflect avoidance of insulin exposure rather than a direct protective effect of GLP-1 RAs. Moreover, although randomized designs mitigate healthy-user bias, participants enrolled in long-term cardiovascular outcome trials may represent a healthier, more adherent subset of patients, potentially limiting generalizability. These factors should be considered when interpreting observed associations.

Given the biological latency of carcinogenesis, longer-term studies are required to determine whether these signals persist or translate into clinically meaningful reductions in cancer risk.

The certainty of evidence, assessed using GRADE methodology, ranged from high for colorectal cancer to moderate for liver cancer to low for pancreatic cancer, reflecting variations in trial consistency and event rates (Nahra et al., 2021; Nauck et al., 2009; Nauck et al., 2014; Nauck et al., 2016; Nino et al., 2018; Pfeffer et al., 2015; Philis‐Tsimikas et al., 2019; Pieber et al., 2019; Pinget et al., 2013; Pi-Sun et al., 2015; Pozzilli et al., 2017; Pratley et al., 2019; Pratley et al., 2010). These gradings temper the strength of our conclusions and highlight the need for continued investigation. Despite the strength of this meta-analysis including a large cumulative sample size, exclusive use of RCTs, and comprehensive subgroup analysis some limitations must be acknowledged. First, cancer was not a primary endpoint in most included trials, which may have led to underreporting or misclassification. Second, the follow-up durations in several trials were insufficient to assess long-term carcinogenesis. Third, individual-level patient data (IPD) were not available, limiting our ability to adjust for important confounders such as smoking, alcohol intake, family history, and genetic risk.

Since most studies focused on liraglutide and semaglutide, the outcomes are most relevant to these drugs, even though the pooled analysis considered all data from the GLP-1 RA class. Although class effects were observed, variations in molecular structure, receptor affinity, and pharmacokinetics may contribute to differential outcomes among GLP-1 RAs. Larger trials directly comparing individual analogs with cancer endpoints are needed to confirm these findings.

### 4.1 Future clinical and research applications

#### 4.1.1 Future research directions

While reassuring data are accumulating about the safety profile of GLP-1 receptor agonists (GLP-1 RAs) used for diabetes and obesity management, there are still significant gaps in our understanding that justifies the need for well-designed multidisciplinary translational and clinical studies.

1. Compound-Specific Oncologic Effects

As highlighted in this meta-analysis, no conclusive association between the use of GLP-1 RAs and an increase in gastrointestinal cancers has been established. However, the differences between agents remain unexplored. There is a need for large-scale observational studies or head-to-head randomized trials that evaluate whether liraglutide, semaglutide, dulaglutide, and newer dual agonists such as tirzepatide offer different cancer risks or benefits due to their structural differences.

2. Longitudinal Studies with Extended Follow-Up

Based on our meta-analysis, most RCTs had a maximum follow-up period of 5 years. This duration may not be sufficient for identifying slow-growing or late-onset cancers. Trials alongside observational cohorts should prioritize longer follow-up periods beyond 10 years to monitor long-term safety signals.

3. Mechanistic and Molecular Pathway Studies

Several biological pathways have been proposed to explain a potential association between GLP-1 receptor agonists and cancer risk reduction, including improved insulin sensitivity, attenuation of systemic inflammation, modulation of gut microbiota composition, and downstream effects on proliferative signaling pathways such as the mammalian target of rapamycin (mTOR). However, it is critical to emphasize that the clinical data included in this meta-analysis do not directly demonstrate activation or inhibition of these pathways at the tissue or molecular level. Rather, these mechanisms remain biologically plausible hypotheses derived primarily from preclinical and translational studies. Accordingly, mechanistic interpretations should not be construed as confirmed clinical mechanisms but as explanatory frameworks requiring validation in mechanistic human studies. These potential pathways are summarized in Figure 5 and warrant further translational research for validation.

4. Individual-Level Risk Stratification

It is important to address the concerns of rare, prolonged use of GLP-1 RA for targeted patients who may benefit or suffer from prolonged exposure. Targeted research for patients with type 2 diabetes mellitus (T2DM) and obesity should focus on defining cancer risk epigenomic and genomic signatures.

5. Post-Marketing Surveillance and Real-World Data Integration

Programs like U.S. Food and Drug Administration (FDA) Sentinel and EudraVigilance serve as national and international data warehouses that support expending surveillance of rare oncologic adverse events associated with neoplasms of oncologic proportions. These programs must be linked with cancer registries alongside electronic health records to enhance external validity for detecting seriously debilitating outcomes that are infrequently occurring.

#### 4.1.2 Clinical applications and implications

This meta-analysis brings to light various clinically relevant aspects that must be considered from a public health perspective:

1. Reassurance in Clinical Decision-Making

As part of our results, it is evident that GLP-1 RAs do not increase the risk for gastrointestinal cancers and may even have protective effects for colorectal and liver cancers. These findings are comforting as clinicians can now safely use these agents in patients with metabolic syndrome and those at a higher cardiovascular risk. These findings support the continued use of GLP-1 receptor agonists for approved metabolic indications, while acknowledging that the certainty of evidence varies by cancer site. In particular, conclusions regarding pancreatic cancer risk remain limited by low GRADE certainty, wide confidence intervals, and heterogeneous outcome definitions. Clinical decisions should therefore remain individualized, and continued long-term surveillance is warranted.

2. Guiding Regulatory and Labeling Policies
  Drug warnings related to GLP-1 RA-associated neoplasms continue to be included in some regions. Our analysis supports these warnings being lifted, especially for semaglutide and liraglutide, which have been studied in large-scale, diverse population treatment-emergent (TE) crossover studies.
3. Oncology–Metabolic Interface

The linkage between metabolic disorders and oncology is becoming more apparent. GLP-1 RAs may provide additional therapeutic benefits beyond glycemic control due to their multifaceted metabolic and anti-inflammatory properties. Their potential repositioning as adjuncts for cancer prevention or treatment in high-risk populations like those with obesity or non-alcoholic fatty liver disease (NAFLD) needs to be investigated in translational oncology.

4. Patient Counseling and Shared Decision-Making

Prescribing new medications is often accompanied by concern regarding the risk of developing cancer. These findings equip healthcare professionals with evidence-based strategies to improve discussions around the benefits and perceived risks of therapy, thereby improving adherence and long-term outcomes.

### 4.2 Limitations

This meta-analysis has several limitations. First, individual patient data (IPD) were not available, which restricted our ability to perform subgroup analyses based on detailed patient-level variables such as ethnicity, cancer stage, or medication adherence. Second, while the included studies were all randomized controlled trials, many reported cancer incidences as a secondary or exploratory outcome, potentially leading to underreporting or inconsistent adjudication of events.

Third, residual confounding from unmeasured factors such as smoking status, alcohol intake, family history of cancer, and dietary patterns may have influenced the observed associations but could not be adjusted for in this aggregate-level analysis. Fourth, the median follow-up duration in several trials was less than 5 years, which may not be sufficient to detect late-onset malignancies, particularly for solid tumors.

Fifth, in some studies, hazard ratios were derived from time-to-event data or calculated from secondary endpoints, which may have introduced variability in effect estimates. Additionally, geographic bias may limit generalizability: a potential geographic bias exists, as a substantial proportion of the included trials were conducted in North America and Europe, limiting generalizability to underrepresented populations in Asia, Africa, and South America.

An important limitation of the available evidence is the relatively short duration of follow-up in many included trials. As most RCTs were designed to evaluate metabolic or cardiovascular outcomes rather than cancer endpoints, their follow-up periods may be insufficient to fully capture the latency of solid tumor development. Consequently, observed reductions in cancer incidence—particularly for colorectal and liver cancers should be interpreted with caution and cannot be assumed to reflect long-term cancer prevention.

Furthermore, the biological latency of solid gastrointestinal malignancies often exceeds the follow-up duration of available randomized trials. Although large studies such as the SELECT trial provide important safety data, a median follow-up of approximately 40 months is insufficient to capture late-onset oncogenesis. As a result, the absence of increased cancer incidence in short-to mid-term follow-up cannot be equated with long-term oncological safety.

Finally, although the risk of bias was generally low across studies, sponsorship bias cannot be ruled out given that a substantial proportion of the included RCTs were industry-funded.

Importantly, these findings should not be extrapolated to newer dual incretin agonists such as tirzepatide. Dual GLP-1/GIP receptor agonists possess distinct pharmacodynamic and metabolic profiles, and long-term oncological safety data for these agents remain limited.

## 5 Conclusion

In conclusion, this meta-analysis provides robust evidence that GLP-1 receptor agonists do not significantly increase the risk of gastrointestinal cancers in patients with T2DM and obesity. On the contrary, GLP-1 RAs may confer protective effects against colorectal and liver cancers, likely due to their weight loss and anti-inflammatory properties. These findings support the continued use of GLP-1 RAs in clinical practice, but further research is needed to fully understand their long-term oncological safety, particularly in relation to non-gastrointestinal cancers and rare malignancies such as pancreatic cancer. The rising global prevalence of T2DM and obesity makes understanding the full range of benefits and risks associated with GLP-1 RAs essential for optimizing treatment strategies in these populations. These results apply specifically to GLP-1 receptor agonists evaluated in randomized controlled trials and should not be extended to dual agonist therapies in the absence of dedicated long-term cancer outcome data.

## Data Availability

The raw data supporting the conclusions of this article will be made available by the authors, without undue reservation.

## References

[B1] Ahmann A. Rodbard H. W. Rosenstock J. Lahtela J. T. de Loredo L. Tornøe K. (2015). Efficacy and safety of liraglutide *versus* placebo added to basal insulin analogues (with or without metformin) in patients with type 2 diabetes: a randomized, placebo-controlled trial. Diabetes Obes. Metab. 17, 1056–1064. 10.1111/dom.12539 26179619 PMC5054929

[B2] Ahrén B. Leguizamo Dimas A. Miossec P. Saubadu S. Aronson R. (2013). Efficacy and safety of lixisenatide once-daily morning or evening injections in type 2 diabetes inadequately controlled on metformin (GetGoal-M). Diabetes Care 36, 2543–2550. 23536584 10.2337/dc12-2006 PMC3747937

[B3] Ahrén B. Johnson S. L. Stewart M. Cirkel D. T. Yang F. Perry C. (2014). HARMONY 3: 104-week randomized, double-blind, placebo- and active-controlled trial assessing the efficacy and safety of albiglutide compared with placebo, sitagliptin, and glimepiride in patients with type 2 diabetes taking metformin. Diabetes Care 37, 2141–2148. 10.2337/dc14-0024 24898304

[B4] Araki E. Inagaki N. Tanizawa Y. Oura T. Takeuchi M. Imaoka T. (2015). Efficacy and safety of once-weekly dulaglutide in com-bination with sulphonylurea and/or biguanide compared with once-daily insulin glargine in Japanese patients with type 2 di-abetes: a randomized, open-label, phase III, non-inferiority study. Diabetes Obes. Metab. 17, 994–1002. 10.1111/dom.12540 26179754 PMC5042081

[B5] Aroda V. R. Rosenstock J. Wysham C. Unger J. Bellido D. González-Gálvez G. (2016). Efficacy and safety of LixiLan, a titratable fixed-ratio combination of insulin glargine plus lixisenatide in type 2 diabetes inadequately controlled on basal insulin and metformin: the LixiLan-L randomized trial. Diabetes Care 39, 1972–1980. 10.2337/dc16-1495 27650977

[B6] Aroda V. R. Bain S. C. Cariou B. Piletič M. Rose L. Axelsen M. (2017). Efficacy and safety of once-weekly semaglutide *versus* once-daily insulin glargine as add-on to metformin (with or without sulfonylureas) in insulin-naive patients with type 2 diabetes (SUSTAIN 4): a randomised, open-label, parallel-group, multicentre, multinational, phase 3a trial. Lancet Diabetes Endocrinol. 5, 355–366. 10.1016/S2213-8587(17)30085-2 28344112

[B7] Aroda V. R. González-Galvez G. Grøn R. Halladin N. Haluzík M. Jermendy G. (2019). Durability of insulin degludec plus liraglutide *versus* insulin glargine U100 as initial injectable therapy in type 2 diabetes (DUAL VIII): a multicentre, open-label, phase 3b, randomised controlled trial. Lancet Diabetes Endocrinol. 7, 596–605. 10.1016/S2213-8587(19)30184-6 31189519

[B8] Aroda V. R. Frias J. P. Ji L. Niemoeller E. Nguyên‐Pascal M. L. Denkel K. (2023). Efficacy and safety of once-weekly efpeglenatide in people with suboptimally controlled type 2 diabetes: the AMPLITUDE-D, AMPLITUDE-L and AMPLITUDE-S randomized controlled trials. Diabetes Obes. Metab. 25, 2084–2095. 10.1111/dom.15079 37013892

[B9] Blonde L. Jendle J. Gross J. Woo V. Jiang H. Fahrbach J. L. (2015). Once-weekly dulaglutide *versus* bedtime insulin glargine, both in combination with prandial insulin lispro, in patients with type 2 diabetes (AWARD-4): a randomised, open-label, phase 3, non-inferiority study. Lancet 385, 2057–2066. 10.1016/S0140-6736(15)60936-9 26009229

[B10] Bolli G. B. Munteanu M. Dotsenko S. Niemoeller E. Boka G. Wu Y. (2014). Efficacy and safety of lixisenatide once daily vs. placebo in people with Type 2 diabetes insufficiently controlled on metformin (GetGoal-F1). Diabet. Med. 31, 176–184. 10.1111/dme.12328 24117597

[B11] Buse J. B. Vilsbøll T. Thurman J. Blevins T. C. Langbakke I. H. Bøttcher S. G. (2014). Contribution of liraglutide in the fixed-ratio combination of insulin degludec and liraglutide (IDegLira). Diabetes Care 37, 2926–2933. 10.2337/dc14-0785 25114296

[B12] Buse J. B. Christensen H. N. Harty B. J. Mitchell J. Soule B. P. Zacherle E. (2023). Study design and baseline profile for adults with type 2 diabetes in the once-weekly subcutaneous SEmaglutide randomized PRAgmatic (SEPRA) trial. BMJ Open Diabetes Res. 11, e003206. 10.1136/bmjdrc-2022-003206 PMC10163526 37137527

[B14] D'Alessio D. Häring H. Charbonnel B. de Pablos‐Velasco P. Candelas C. Dain M. (2015). Comparison of insulin glargine and liraglutide added to oral agents in patients with poorly controlled type 2 diabetes. Diabetes Obes. Metab. 17, 170–178. 10.1111/dom.12406 25359159

[B15] Dankner R. Cheung N. W. Keinan-Boker L. (2024). Pancreatic cancer risk in patients treated with GLP-1 receptor agonists: a 9-year nationwide cohort study. J. Clin. Endocrinol. Metab. 109, 234–243. 10.1001/jamanetworkopen.2023.50408

[B16] Davies M. J. Bergenstal R. Bode B. Kushner R. F. Lewin A. Skjøth T. V. (2015). Efficacy of liraglutide for weight loss among patients with type 2 diabetes: the SCALE diabetes randomized clinical trial. JAMA 314, 687–699. 10.1001/jama.2015.9676 26284720

[B17] Davies M. J. Bain S. C. Atkin S. L. Rossing P. Scott D. Shamkhalova M. S. (2016). Efficacy and safety of liraglutide *versus* placebo as Add-on to glucose-lowering therapy in patients with type 2 diabetes and moderate renal impairment (LIRA-RENAL): a randomized clinical trial. Diabetes Care 39, 222–230. 10.2337/dc14-2883 26681713

[B18] Davies M. Færch L. Jeppesen O. K. Pakseresht A. Pedersen S. D. Perreault L. (2021). Semaglutide 2·4 mg once a week in adults with overweight or obesity, and type 2 diabetes (STEP 2): a randomised, double-blind, double-dummy, placebo-controlled, phase 3 trial. Lancet 397, 971–984. 10.1016/S0140-6736(21)00213-0 33667417

[B19] Del Prato S. E Kahn S. Pavo I. Weerakkody G. J. Yang Z. Doupis J. (2021). Tirzepatide *versus* insulin glargine in type 2 diabetes and increased cardiovascular risk (SURPASS-4): a randomised, open-label, parallel-group, multicentre, phase 3 trial. Lancet 398, 1811–1824. 10.1016/S0140-6736(21)02188-7 34672967

[B20] Diamant M. Nauck M. A. Shaginian R. Malone J. K. Cleall S. Reaney M. (2014a). Glucagon-like peptide 1 receptor agonist or bolus insulin with optimized basal insulin in type 2 diabetes. Diabetes Care 37, 2763–2773. 10.2337/dc14-0876 25011946

[B21] Diamant M. Van Gaal L. Guerci B. Stranks S. Han J. Malloy J. (2014b). Exenatide once weekly *versus* insulin glargine for type 2 diabetes (DURATION-3): 3-year results of an open-label randomised trial. Lancet Diabetes Endocrinol. 2, 464–473. 10.1016/S2213-8587(14)70029-4 24731672

[B13] Figlioli G. Piovani D. Peppas S. Pugliese N. Hassan C. Repici A. (2024). Glucagon-like peptide-1 receptor agonists and risk of gastrointestinal cancers: a systematic review and meta-analysis of randomized controlled trials. Pharmacol. Res. 208, 107401. 10.1016/j.phrs.2024.107401 39251099

[B22] Frías J. P. Guja C. Hardy E. Ahmed A. Dong F. Öhman P. (2016). Exenatide once weekly plus dapagliflozin once daily versus exenatide or dapagliflozin alone in patients with type 2 diabetes inadequately controlled with metformin monotherapy (DURATION-8): a 28 week, multicentre, double-blind, phase 3, randomised controlled trial. Lancet Diabetes Endocrinol. 4, 1004–1016. 10.1016/s2213-8587(16)30267-4 27651331

[B23] Gallwitz B. Böhmer M. Segiet T. Mölle A. Milek K. Becker B. (2011). Exenatide twice daily *versus* premixed insulin aspart 70/30 in metformin-treated patients with type 2 diabetes: a randomized 26-week study on glycemic control and hypoglycemia. Diabetes Care 34, 604–606. 10.2337/dc10-1900 21285388 PMC3041190

[B24] Gallwitz B. Guzman J. Dotta F. Guerci B. Simó R. Basson B. R. (2012). Ex-enatide twice daily *versus* glimepiride for prevention of glycaemic deterioration in patients with type 2 diabetes with metformin failure (EUREXA): an open-label, randomised controlled trial. Lancet 379, 2270–2278. 10.1016/S0140-6736(12)60479-6 22683137

[B25] Gao L. Lee B. W. Chawla M. Kim J. Huo L. Du L. (2023). Tirzepatide *versus* insulin glargine as second-line or third-line therapy in type 2 diabetes in the Asia-Pacific region: the SURPASS-AP-Combo trial. Nat. Med. 29, 1500–1510. 10.1038/s41591-023-02344-1 37231074

[B26] Garber A. Henry R. Ratner R. Garcia-Hernandez P. A. Rodriguez-Pattzi H. Olvera-Alvarez I. (2009). Liraglutide *versus* glimepiride monotherapy for type 2 diabetes (LEAD-3 Mono): a randomised, 52-week, phase III, double-blind, parallel-treatment trial. Lancet 373, 473–481. 10.1016/S0140-6736(08)61246-5 18819705

[B27] Garvey W. T. Birkenfeld A. L. Dicker D. Mingrone G. Pedersen S. D. Satylganova A. (2020). Efficacy and safety of liraglutide 3.0 mg in individuals with overweight or obesity and type 2 diabetes treated with basal insulin: the SCALE insulin randomized controlled trial. Diabetes Care 43, 1085–1093. 10.2337/dc19-1745 32139381 PMC7171937

[B28] Gerstein H. C. Colhoun H. M. Dagenais G. R. Diaz R. Lakshmanan M. Pais P. (2019). Dulaglutide and cardiovascular outcomes in type 2 diabetes (REWIND): a double-blind, randomised place-bo-controlled trial. Lancet 394, 121–130. 10.1016/S0140-6736(19)31149-3 31189511

[B29] Gerstein H. C. Sattar N. Rosenstock J. Ramasundarahettige C. Pratley R. Lopes R. D. (2021). Cardiovascular and renal outcomes with efpeglenatide in type 2 diabetes. N. Engl. J. Med. 385, 896–907. 10.1056/NEJMoa2108269 34215025

[B30] Gough S. C. L. Bode B. Woo V. Rodbard H. W. Linjawi S. Poulsen P. (2014). Efficacy and safety of a fixed-ratio combination of insulin degludec and liraglutide (IDegLira) compared with its components given alone: results of a phase 3, open-label, randomised, 26-week, treat-to-target trial in insulin-naive patients with type 2 d. Lancet Diabetes Endocrinol. 2, 885–893. 10.1016/S2213-8587(14)70174-3 25190523

[B31] Hernandez A. F. Green J. B. Janmohamed S. D'Agostino R. B. Granger C. B. Jones N. P. (2018). Albiglutide and cardiovascular outcomes in patients with type 2 diabetes and cardio-vascular disease (Harmony Outcomes): a double-blind, randomised placebo-controlled trial. Lancet 392, 1519–1529. 10.1016/S0140-6736(18)32261-X 30291013

[B32] Holman R. R. Bethel M. A. Mentz R. J. Thompson V. P. Lokhnygina Y. Buse J. B. (2017). Effects of once-weekly exenatide on cardiovascular outcomes in type 2 diabetes. N. Engl. J. Med. 377, 1228–1239. 10.1056/NEJMoa1612917 28910237 PMC9792409

[B33] Home P. D. Shamanna P. Stewart M. Yang F. Miller M. Perry C. (2015). Efficacy and tolerability of albiglutide *versus* placebo or pioglitazone over 1 year in people with type 2 diabetes currently taking metformin and glimepiride: HARMONY 5. Diabetes. Obes. Metab. 17, 179–187. 10.1111/dom.12414 25406730

[B34] Husain M. Birkenfeld A. L. Donsmark M. Dungan K. Eliaschewitz F. G. Franco D. R. (2019). Oral semaglutide and cardio-vascular outcomes in patients with type 2 diabetes. N. Engl. J. Med. 381, 841–851. 10.1056/NEJMoa1901118 31185157

[B35] Jastreboff A. M. Aronne L. J. Ahmad N. N. Wharton S. Connery L. Alves B. (2022). Tirzepatide once weekly for the treatment of obesity. N. Engl. J. Med. 387, 205–216. 10.1056/NEJMoa2206038 35658024

[B36] Kadowaki T. Namba M. Imaoka T. Yamamura A. Goto W. Boardman M. K. (2011). Improved glycemic control and reduced bodyweight with exenatide: a double-blind, randomized, phase 3 study in Japanese patients with suboptimally con-trolled type 2 diabetes over 24 weeks. J. Diabetes Investig. 2, 210–217. 10.1111/j.2040-1124.2010.00084.x 24843486 PMC4014921

[B37] Kadowaki T. Isendahl J. Khalid U. Lee S. Y. Nishida T. Ogawa W. (2022). Semaglutide once a week in adults with overweight or obesity, with or without type 2 diabetes in an east Asian population (STEP 6): a randomised, double-blind, double-dummy, placebo-controlled, phase 3a trial. Lancet Diabetes Endocrinol. 10, 193–206. 10.1016/S2213-8587(22)00008-0 35131037

[B38] Kaku K. Rasmussen M. F. Nishida T. Seino Y. (2011). Fifty-two-week, randomized, multicenter trial to compare the safety and efficacy of the novel glucagon-like peptide-1 analog liraglutide vs. glibenclamide in patients with type 2 diabetes. J. Diabetes Investig. 2, 441–447. 10.1111/j.2040-1124.2011.00128.x 24843528 PMC4014903

[B39] Kaku K. Kiyosue A. Ono Y. Shiraiwa T. Kaneko S. Nishijima K. (2016). Liraglutide is effective and well tolerated in combination with an oral antidiabetic drug in Japanese patients with type 2 diabetes: a randomized, 52-week, open-label, parallel-group trial. J. Diabetes Investig. 7, 76–84. 10.1111/jdi.12367 26816604 PMC4718097

[B40] Kaku K. Yamada Y. Watada H. Abiko A. Nishida T. Zacho J. (2018). Safety and efficacy of once-weekly semaglutide vs additional oral antidiabetic drugs in Japanese people with inadequately controlled type 2 diabetes: a randomized trial. Diabetes Obes. Metab. 20, 1202–1212. 10.1111/dom.13218 29322610 PMC5969242

[B41] Kaku K. Araki E. Tanizawa Y. Ross Agner B. Nishida T. Ranthe M. (2019). Superior efficacy with a fixed-ratio com-bination of insulin degludec and liraglutide (IDegLira) compared with insulin degludec and liraglutide in insulin-naïve Japanese patients with type 2 diabetes in a phase 3, open-label, randomized trial. Diabetes Obes. Metab. 21, 2674–2683. 10.1111/dom.13856 31407845 PMC6899795

[B42] Kellerer M. Kaltoft M. S. Lawson J. Nielsen L. L. Strojek K. Tabak Ö. (2022). Effect of once-weekly semaglutide *versus* thrice-daily insulin aspart, both as add-on to metformin and optimized insulin glargine treatment in participants with type 2 diabetes (SUSTAIN 11): a randomized, open-label, multinational, phase 3b trial. Diabetes Obes. Metab. 24, 1788–1799. 10.1111/dom.14765 35546450 PMC9545869

[B43] Knop F. K. Aroda V. R. Vale R. D. D. Holst-Hansen T. Laursen P. N. Rosenstock J. (2023). Oral semaglutide 50 mg taken once per day in adults with overweight or obesity (OASIS 1): a randomised, double-blind, place-bo-controlled, phase 3 trial. Lancet 402, 705–719. 10.1016/S0140-6736(23)01185-6 37385278

[B44] Le Roux C. W. Astrup A. Fujioka K. Greenway F. Lau D. C. Van Gaal L. (2017). 3 years of liraglutide *versus* placebo for type 2 diabetes risk reduction and weight management in individuals with prediabetes: a randomised, double-blind trial. Lancet 389, 1399–1409. 10.1016/S0140-6736(17)30069-7 28237263

[B45] Leiter L. A. Carr M. C. Stewart M. Jones-Leone A. Scott R. Yang F. (2014). Efficacy and safety of the once-weekly GLP-1 receptor agonist albiglutide *versus* sitagliptin in patients with type 2 diabetes and renal impairment: a randomized phase III study. Diabetes Care 37, 2723–2730. 10.2337/dc13-2855 25048383

[B46] Lingvay I. Pérez Manghi F. García-Hernández P. Norwood P. Lehmann L. Tarp-Johansen M. J. (2016). Effect of insulin glargine Up-titration vs insulin Degludec/Liraglutide on glycated hemoglobin levels in patients with uncontrolled type 2 diabetes: the DUAL V randomized clinical trial. JAMA 315, 898–907. 10.1001/jama.2016.1252 26934259

[B47] Lingvay I. Desouza C. V. Lalic K. S. Rose L. Hansen T. Zacho J. (2018). A 26-Week randomized controlled trial of semaglutide once daily *versus* liraglutide and placebo in patients with type 2 diabetes suboptimally controlled on diet and exercise with or without metformin. Diabetes Care 41, 1926–1937. 10.2337/dc17-2381 30026333

[B50] Ludvik B. Giorgino F. Jódar E. Frias J. P. Fernández Landó L. Brown K. (2021). Once-weekly tirzepatide versus once-daily insulin degludec as add-on to metformin with or without SGLT2 inhibitors in patients with type 2 diabetes (SURPASS-3): a randomised, open-label, parallel-group, phase 3 trial. Lancet. 14, 398 (10300), 583–598. 10.1016/s0140-6736(21)01443-4 34370970

[B48] Marso S. P. Bain S. C. Consoli A. Eliaschewitz F. G. Jódar E. Leiter L. A. (2016a). Semaglutide and cardiovascular outcomes in patients with type 2 diabetes. N. Engl. J. Med. 375, 1834–1844. 10.1056/NEJMoa1607141 27633186

[B49] Marso S. P. Daniels G. H. Brown-Frandsen K. Kristensen P. Mann J. F. E. Nauck M. A. (2016b). Liraglutide and cardiovascular outcomes in type 2 diabetes. N. Engl. J. Med. 375, 311–322. 10.1056/nejmoa1603827 27295427 PMC4985288

[B51] Miyagawa J. Odawara M. Takamura T. Iwamoto N. Takita Y. Imaoka T. (2015). Once-weekly glucagon-like peptide-1 receptor agonist dulaglutide is non-inferior to once-daily liraglutide and superior to placebo in Japanese patients with type 2 diabetes: a 26-week randomized phase III study. Diabetes Obes. Metab. 17, 974–983. 10.1111/dom.12534 26179187 PMC5042083

[B52] Nahra R. Wang T. Gadde K. M. Oscarsson J. Stumvoll M. Jermutus L. (2021). Effects of cotadutide on metabolic and hepatic parameters in adults with overweight or obesity and type 2 diabetes: a 54-Week randomized phase 2b Study. Diabetes Care 44, 1433–1442. 10.2337/dc20-2151 34016612 PMC8247525

[B53] Nauck M. Frid A. Hermansen K. Shah N. S. Tankova T. Mitha I. H. (2009). Efficacy and safety comparison of liraglutide, glimepiride, and placebo, all in combination with metformin, in type 2 diabetes: the LEAD (liraglutide effect and action in diabetes)-2 study. Diabetes Care 32, 84–90. 10.2337/dc08-1355 18931095 PMC2606836

[B54] Nauck M. Weinstock R. S. Umpierrez G. E. Guerci B. Skrivanek Z. Milicevic Z. (2014). Efficacy and safety of dulaglutide *versus* sitagliptin after 52 weeks in type 2 diabetes in a randomized controlled trial (AWARD-5). Diabetes Care 37, 2149–2158. 10.2337/dc13-2761 24742660 PMC4113177

[B55] Nauck M. A. Stewart M. W. Perkins C. Jones-Leone A. Yang F. Perry C. (2016). Efficacy and safety of once-weekly GLP-1 receptor agonist albiglutide (HARMONY 2): 52 week primary endpoint results from a randomised, pla-cebo-controlled trial in patients with type 2 diabetes mellitus inadequately controlled with diet and exercise. Diabetologia 59, 266–274. 10.1007/s00125-015-3795-1 26577795 PMC4705137

[B56] Nino A. Okuda I. Wilson T. H. Yue L. Nakajima H. Tsuboi M. (2018). Weekly glucagon-like peptide-1 receptor agonist albiglutide as monotherapy improves glycemic parameters in Japanese patients with type 2 diabetes mellitus: a ran-domized, double-blind, placebo-controlled study. J. Diabetes Investig. 9, 558–566. 10.1111/jdi.12749 28921915 PMC5934244

[B57] Pfeffer M. A. Claggett B. Diaz R. Dickstein K. Gerstein H. C. Køber L. V. (2015). Lixisenatide in patients with type 2 diabetes and acute coronary syndrome. N. Engl. J. Med. 373, 2247–2257. 10.1056/NEJMoa1509225 26630143

[B58] Philis‐Tsimikas A. Billings L. K. Busch R. Portillo C. M. Sahay R. Halladin N. (2019). Superior efficacy of insulin degludec/liraglutide *versus* insulin glargine U100 as add-on to sodium-glucose co-transporter-2 inhibitor therapy: a randomized clinical trial in people with uncontrolled type 2 diabetes. Diabetes Obes. Metab. 21, 1399–1408. 10.1111/dom.13666 30761720 PMC6593861

[B59] Pi-Sunyer X. Astrup A. Fujioka K. Greenway F. Halpern A. Krempf M. (2015). A randomized, Controlled Trial of 3.0 mg of Liraglutide in Weight Management. N. Engl. J. Med. 373, 11–22. 10.1056/NEJMoa1411892 26132939

[B60] Pieber T. R. Bode B. Mertens A. Cho Y. M. Christiansen E. Hertz C. L. (2019). Efficacy and safety of oral semaglutide with flexible dose adjustment *versus* sitagliptin in type 2 diabetes (PIONEER 7): a multicentre, open-label, randomised, phase 3a trial. Lancet Diabetes Endocrinol. 7, 528–539. 10.1016/S2213-8587(19)30194-9 31189520

[B61] Pinget M. Goldenberg R. Niemoeller E. Muehlen-Bartmer I. Guo H. Aronson R. (2013). Efficacy and safety of lixisenatide once daily *versus* placebo in type 2 diabetes insufficiently controlled on pioglitazone (GetGoal-P). Diabetes Obes. Metab. 15, 1000–1007. 10.1111/dom.12121 23627775

[B62] Pozzilli P. Norwood P. Jódar E. Davies M. J. Ivanyi T. Jiang H. (2017). Placebo-controlled, randomized trial of the addition of once-weekly glucagon-like peptide-1 receptor agonist dulaglutide to titrated daily insulin glargine in patients with type 2 dia-betes (AWARD-9). Diabetes Obes. Metab. 19, 1024–1031. 10.1111/dom.12937 28294499

[B63] Pratley R. E. Nauck M. Bailey T. Montanya E. Cuddihy R. Filetti S. (2010). Liraglutide *versus* sitagliptin for patients with type 2 diabetes who did not have adequate glycaemic control with metformin: a 26-week, randomised, parallel-group, open-label trial. Lancet 375, 1447–1456. 10.1016/S0140-6736(10)60307-8 20417856

[B64] Pratley R. Amod A. Hoff S. T. Kadowaki T. Lingvay I. Nauck M. (2019). Oral semaglutide *versus* subcutaneous liraglutide and placebo in type 2 diabetes (PIONEER 4): a randomised, double-blind, phase 3a trial. Lancet 394, 39–50. 10.1016/S0140-6736(19)31271-1 31186120

