# Supplementary material for: Reassessing cancer risk with GLP-1 receptor agonists: a comprehensive meta-analysis of gastrointestinal malignancies
Source: Front Pharmacol. 2026 Feb 10;17:1736380. doi: 10.3389/fphar.2026.1736380 (PMC12929497; doi:10.3389/fphar.2026.1736380)
Supplement: Supplementary file 1 [file Table1.docx]

# Supplementary Table S1. Full Electronic Search Strategies

This table presents the full reproducible electronic search strategies used for PubMed/MEDLINE, Embase, and Scopus, conducted in August 2024, in line with PRISMA-S guidelines. All strategies were designed to capture randomized controlled trials (RCTs) evaluating GLP-1 receptor agonists and gastrointestinal cancers.

| Database | Search Strategy (verbatim) | Limits/Notes |
| --- | --- | --- |
| PubMed/MEDLINE | ("glucagon-like peptide-1 receptor agonist"[tiab] OR "GLP-1 receptor agonist"[tiab] OR "glp1"[tiab] OR exenatide[tiab] OR lixisenatide[tiab] OR liraglutide[tiab] OR semaglutide[tiab] OR dulaglutide[tiab] OR efpeglenatide[tiab] OR albiglutide[tiab]) AND (colorectal[tiab] OR colon[tiab] OR rectal[tiab] OR pancreatic[tiab] OR pancreas[tiab] OR gastric[tiab] OR stomach[tiab] OR liver[tiab] OR hepatocellular[tiab] OR "gastrointestinal cancer"[tiab] OR "GI cancer"[tiab] OR neoplasm*[tiab] OR carcinoma*[tiab] OR cancer*[tiab]) AND (randomized[tiab] OR randomised[tiab] OR "randomized controlled trial"[Publication Type] OR "controlled clinical trial"[Publication Type] OR "randomized controlled trials as topic"[MeSH Terms]) NOT (animals[mh] NOT humans[mh]) | Humans only; no language or date restrictions; last searched August 15, 2024. |
| Embase (Ovid) | 1. 'glucagon like peptide 1 receptor agonist'/exp OR exenatide:ti,ab OR liraglutide:ti,ab OR semaglutide:ti,ab OR dulaglutide:ti,ab OR lixisenatide:ti,ab 2. 'gastrointestinal cancer'/exp OR colorectal:ti,ab OR pancreatic:ti,ab OR gastric:ti,ab OR liver:ti,ab OR neoplasm*:ti,ab OR carcinoma*:ti,ab OR cancer*:ti,ab 3. 'randomized controlled trial'/exp OR random*:ti,ab OR placebo*:ti,ab 4. 1 AND 2 AND 3 | Humans only; no date restrictions; last searched August 15, 2024. |
| Scopus | TITLE-ABS-KEY("GLP-1 receptor agonist" OR exenatide OR liraglutide OR semaglutide OR dulaglutide OR lixisenatide) AND TITLE-ABS-KEY(colorectal OR pancreatic OR gastric OR liver OR "gastrointestinal cancer" OR neoplasm* OR carcinoma* OR cancer*) AND TITLE-ABS-KEY(random* OR trial OR placebo) | Humans only; Clinical Trial filter applied; last searched August 15, 2024. |

# Supplementary Table S2:PRISMA 2020 Checklist (Final Extended Version)

This final extended checklist has been prepared for the manuscript 'Reassessing Cancer Risk with GLP-1 Receptor Agonists: A Comprehensive Meta-Analysis of Gastrointestinal Malignancies'. It follows PRISMA 2020 reporting standards and includes all required columns.

| Section/Topic | PRISMA 2020 Item | Checklist Item Description | Reported (Yes/No) | Reported on Page/Section |
| --- | --- | --- | --- | --- |
| Title | Identify the report as a systematic review, meta-analysis, or both. | Identify the report as a systematic review, meta-analysis, or both. | Yes | See Methods/Results |
| Abstract | Provide a structured summary including background, objectives, data sources, study eligibility, participants, interventions, methods, results, limitations, conclusions, and registration. | Provide a structured summary including background, objectives, data sources, study eligibility, participants, interventions, methods, results, limitations, conclusions, and registration. | Yes | See Methods/Results |
| Rationale | Describe the rationale for the review in the context of existing knowledge. | Describe the rationale for the review in the context of existing knowledge. | Yes | See Methods/Results |
| Objectives | Provide an explicit statement of the objectives/questions being addressed. | Provide an explicit statement of the objectives/questions being addressed. | Yes | See Methods/Results |
| Eligibility criteria | Specify study characteristics (participants, interventions, comparators, outcomes, study design, length of follow-up) and report characteristics (years considered, language, publication status) used as criteria for eligibility. | Specify study characteristics (participants, interventions, comparators, outcomes, study design, length of follow-up) and report characteristics (years considered, language, publication status) used as criteria for eligibility. | Yes | See Methods/Results |
| Information sources | Describe all information sources (e.g., databases with dates of coverage, contact with study authors) in the search and date last searched. | Describe all information sources (e.g., databases with dates of coverage, contact with study authors) in the search and date last searched. | Yes | See Methods/Results |
| Search strategy | Present full search strategies for at least one database, including limits used, such that it could be repeated. | Present full search strategies for at least one database, including limits used, such that it could be repeated. | Yes | See Methods/Results |
| Selection process | State the process for selecting studies (screening, eligibility, inclusion, reasons for exclusion). | State the process for selecting studies (screening, eligibility, inclusion, reasons for exclusion). | Yes | See Methods/Results |
| Data collection process | Describe method of data extraction and how discrepancies were resolved. | Describe method of data extraction and how discrepancies were resolved. | Yes | See Methods/Results |
| Data items | List and define all variables for which data were sought. | List and define all variables for which data were sought. | Yes | See Methods/Results |
| Study risk of bias assessment | Describe methods for assessing risk of bias of individual studies. | Describe methods for assessing risk of bias of individual studies. | Yes | See Methods/Results |
| Effect measures | State principal summary measures (e.g., risk ratio, difference in means). | State principal summary measures (e.g., risk ratio, difference in means). | Yes | See Methods/Results |
| Synthesis methods | Describe methods for handling data and combining results of studies, including measures of consistency for meta-analyses. | Describe methods for handling data and combining results of studies, including measures of consistency for meta-analyses. | Yes | See Methods/Results |
| Reporting bias assessment | Describe methods of assessing risk of bias due to missing results in a synthesis (publication bias). | Describe methods of assessing risk of bias due to missing results in a synthesis (publication bias). | Yes | See Methods/Results |
| Certainty assessment | Describe methods used to assess certainty in the body of evidence (e.g., GRADE). | Describe methods used to assess certainty in the body of evidence (e.g., GRADE). | Yes | See Methods/Results |
| Study selection (Results) | Provide numbers of studies screened, assessed, and included, with reasons for exclusions at each stage, ideally with a flow diagram. | Provide numbers of studies screened, assessed, and included, with reasons for exclusions at each stage, ideally with a flow diagram. | Yes | See Methods/Results |
| Study characteristics | Present characteristics for which data were extracted (e.g., study size, PICOS, follow-up period) and provide citations. | Present characteristics for which data were extracted (e.g., study size, PICOS, follow-up period) and provide citations. | Yes | See Methods/Results |
| Risk of bias in studies | Present data on risk of bias for each study and, if available, outcome-level assessment. | Present data on risk of bias for each study and, if available, outcome-level assessment. | Yes | See Methods/Results |
| Results of individual studies | Present for each study: summary data and effect estimates with confidence intervals, ideally with a structured table or plot. | Present for each study: summary data and effect estimates with confidence intervals, ideally with a structured table or plot. | Yes | See Methods/Results |
| Results of syntheses | Present results of each meta-analysis, including confidence/credible intervals and measures of consistency. | Present results of each meta-analysis, including confidence/credible intervals and measures of consistency. | Yes | See Methods/Results |
| Reporting biases | Present assessments of risk of bias due to missing results. | Present assessments of risk of bias due to missing results. | Yes | See Methods/Results |
| Certainty of evidence | Present assessments of certainty for each outcome (e.g., GRADE). | Present assessments of certainty for each outcome (e.g., GRADE). | Yes | See Methods/Results |
| Discussion | Summarize main findings, strengths, limitations, comparison with prior work, and implications for practice/policy/research. | Summarize main findings, strengths, limitations, comparison with prior work, and implications for practice/policy/research. | Yes | See Methods/Results |
| Other information: Registration and protocol | Provide registration information and protocol access. | Provide registration information and protocol access. | Yes | See Methods/Results |
| Support | Describe sources of financial or non-financial support. | Describe sources of financial or non-financial support. | Yes | See Methods/Results |
| Competing interests | Describe authors’ conflicts of interest. | Describe authors’ conflicts of interest. | Yes | See Methods/Results |
| Availability of data, code, and other materials | Report availability of materials/data/code used in the review. | Report availability of materials/data/code used in the review. | Yes | See Methods/Results |
